# Kyste du canal de Nück (hydrocèle de la femme)

**DOI:** 10.11604/pamj.2022.41.337.12532

**Published:** 2022-04-26

**Authors:** Meriem Haloua, Meryem Boubbou

**Affiliations:** 1 Service de Radiologie, CHU Hassan II, Université Sidi Mohammed Ben Abdallah, Fès, Maroc

**Keywords:** Hernie, kyste, chirurgie pédiatrique, Hernia, cyst, paediatric surgery

## Abstract

We here report the case of a 06-year old girl treated for Kawasaki disease admitted with right tender and painful inguinal swelling. Ultrasound images suggested cystic lymphangioma. TDM showed fluid-filled mass in the right iliac fossa (RIF) in contact with the external iliac vessels, the sigmoid colon and a few small bowel loops extending downward from a dehiscence of the inguinal canal into the large lip. It had localized thickening of the wall measuring 5 mm in thickness at the level of the inguinal orifice. The diagnosis of cyst of the canal of Nück (female hydrocele) was retained and surgically confirmed (Figure 1). The cyst of the canal of Nück is caused by incomplete closure of the canal, resulting in fibrous vestigial structure of the peritoneal-vaginal duct which connects the peritoneal cavity with the large lip. This usually closes at birth or during the first month of life, with resultant formation of a cystic lesion bordered by mesothelium, which is predominantly flattened and sometimes hyperplastic, located in the anterosuperior portion of the large lips and/or the inguinal canal. Surgical treatment for cyst of the canal of Nück is the same as for hernia.

## Image en médecine

Une fille de six ans suivie pour maladie de Kawazaki, admise pour tuméfaction inguinale droite molle douloureuse, l´échographie était en faveur d´un lymphangiome kystique. La tomodensitométrie a objectivé une formation liquidienne de la fosse iliaque droite (FIDte) qui vient au contact des vaisseaux iliaques externes, du sigmoïde et de quelques anses grêliques, et s´étend à travers une déhiscence du canal inguinal en direction de la grande lèvre. Elle présente un épaississement localisé de la paroi mesuré à 5 mm d´épaisseur au niveau de l´orifice inguinal (A). Le diagnostic de kyste du canal de Nück (hydrocèle de la femme) a été retenu, et confirmé chirurgicalement (B). Le kyste du canal de Nück est dû à un défaut de fermeture du canal de Nück, qui correspond à une structure fibreuse vestigiale du canal péritonéo-vaginal, qui fait communiquer la cavité péritonéale avec la grande lèvre, qui se ferme normalement à la naissance ou durant le premier mois de la vie, avec formation d´une lésion kystique bordée par du mésothélium souvent aplati parfois hyperplasique, situé dans la partie antéro-supérieure des grandes lèvres et/ou du canal inguinal. Elle est traitée par le chirurgien comme une hernie.

**Figure 1:**
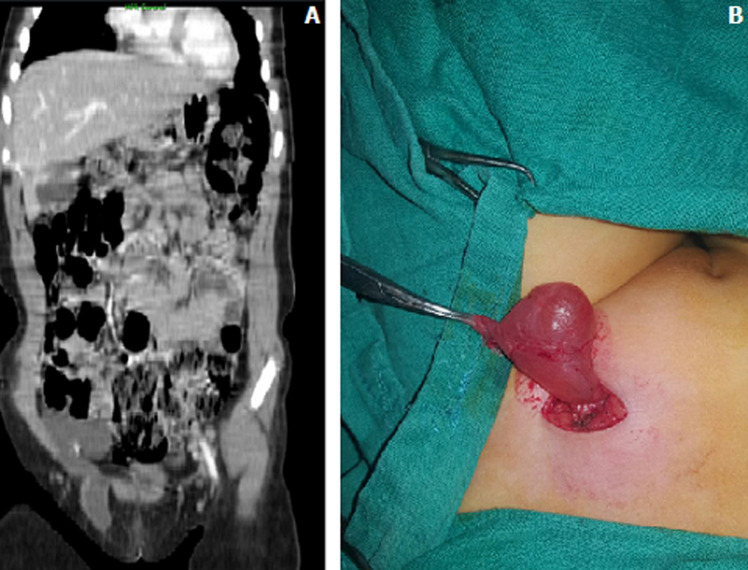
scanner injecté en reconstruction coronale (A), vue per-opératoire (B)

